# A case of malignant gastric glomus tumor and literature review: A case report

**DOI:** 10.1097/MD.0000000000039208

**Published:** 2024-08-09

**Authors:** Jiannan Huang, Chaofeng Yuan, Shaopeng Zhang, Tong Qu, Jian Suo

**Affiliations:** a Department of Gastrointestinal Colorectal Surgery, China-Japan Union Hospital of Jilin University, Changchun, China; b Department of Gastrointestinal Colorectal Surgery, First Hospital of Jilin University, Changchun, China.

**Keywords:** case report, gastric, glomus tumor, malignant, prognosis, therapy

## Abstract

**Rationale:**

Malignant gastric glomus tumor (GGT) is an extremely rare malignant tumor of mesenchymal origin, it affects the patient’s health and even threatens life. Malignant GGT with vascular invasion is even more rarely reported in the available literature without a prognostic study. So, in this case, we report a malignant GGT with vascular invasion and performed a 5-year postoperative follow-up. To the best of our knowledge, we report the first case of malignant GGT with vascular invasion without recurrence 5 years after surgery. This provides examples and lessons for the treatment of malignant GGT with vascular invasion.

**Patient concerns:**

A 49-year-old male was admitted to the hospital with gallbladder stones found on health check. After completing abdominal CT and ultrasound gastroscopy, a mass in the gastric antrum was found.

**Diagnoses:**

The diagnosis of malignant GGT was confirmed by combination of postoperative pathology with positive immunohistochemistry for SMA, vimentin, synaptophysin, H-caldesmon, and calponin, mitosis > 10/50 HPF and moderate-to-severe nuclear atypia.

**Interventions:**

On the 6th day of hospitalization, the patient underwent laparoscopic distal gastrectomy and cholecystectomy.

**Outcomes:**

The patient was discharged successfully 1 week after surgery and was followed up for 5 years without recurrence.

**Conclusion:**

Malignant GGT can be asymptomatic. For malignant GGT without distant metastasis, despite the presence of vascular invasion, negative margin surgery can still be the standard surgical radical treatment.

## 1. Introduction

Glomus tumor (GT) is a rare mesenchymal-derived tumor that occurs mostly in the peripheral soft tissues, especially in the distal extremities. GTs occurring in the stomach are much rarer, Kay et al^[1]^ first reported a case of gastric glomus tumor (GGT) in 1951, and only about 300 reports of GGT can be accessed to date. GGT is more common in women than in men, with a male-to-female ratio of about 1:1.6. The median age of patients is 45 years, and the tumors are mostly located in the gastric antrum.^[2]^ The vast majority of GGTs reported in the literature are benign lesions, and only a very small proportion are malignant GGTs.^[3–13]^ The first case of malignant GGT was reported by Haque et al^[14]^ in 1992. Malignant GGTs have a tendency to recur and metastasize, some malignant GGTs can develop liver, kidney, and brain metastases.^[7,8]^

Case reports of malignant GGT are rare, and malignant GGT with vascular invasion is even more rare. We searched PubMed for accessible case reports of malignant GGT, among which, only 3 cases mentioned vascular invasion in malignant GGT, and 1 article showed microscopic images of vascular invasion in benign GGT.^[8,10–12]^ However, no microscopic picture of vascular invasion in malignant GGT has been demonstrated in the literature yet. Moreover, for malignant GGT with vascular invasion, there is no report of up to 5 years of follow-up without recurrence.

## 2. Case report

**Figure 1.**
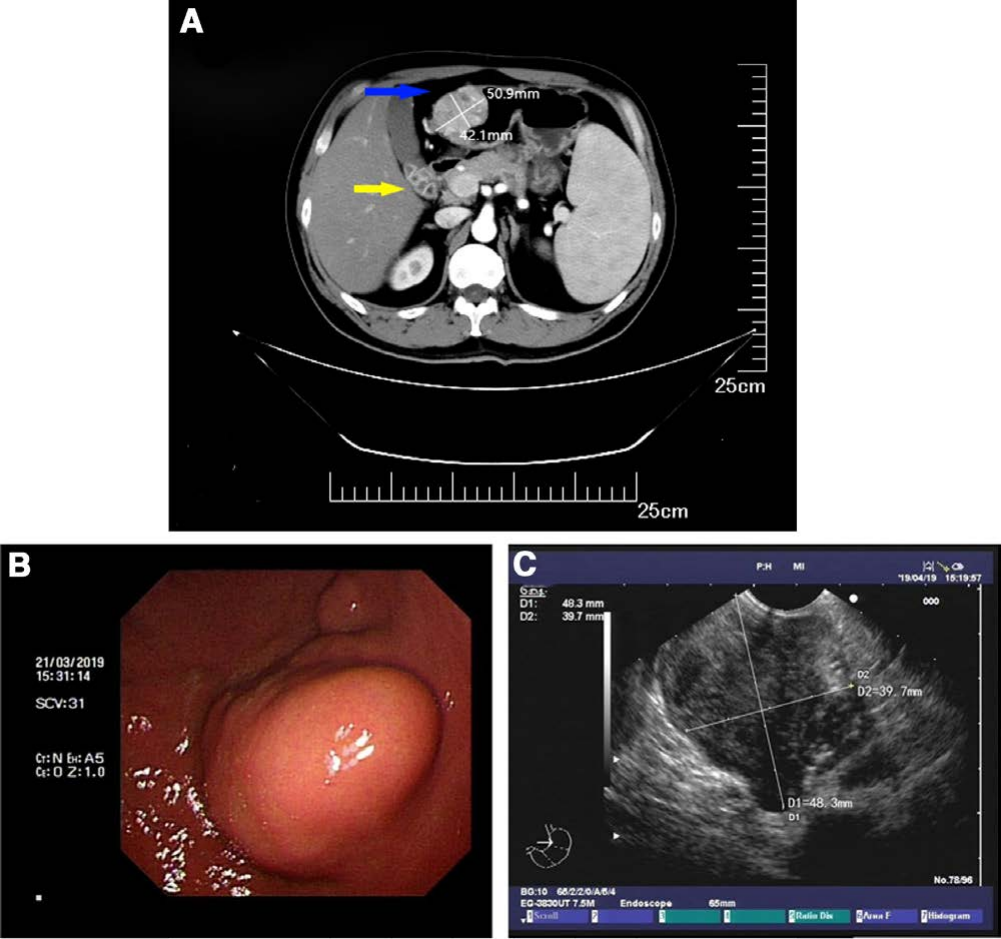
(A) Abdominal CT indicated a 50.9 mm × 42.1 mm mass in the anterior wall of the gastric antrum (blue arrow), with persistent heterogeneous enhancement in the arterial phase and multiple stones in the gallbladder (yellow arrow). (B, C) Ultrasound gastroscopy suggested a 48.3 mm × 39.7 mm hypoechoic mass in the intrinsic muscular layer of the gastric antrum.

**Figure 2.**
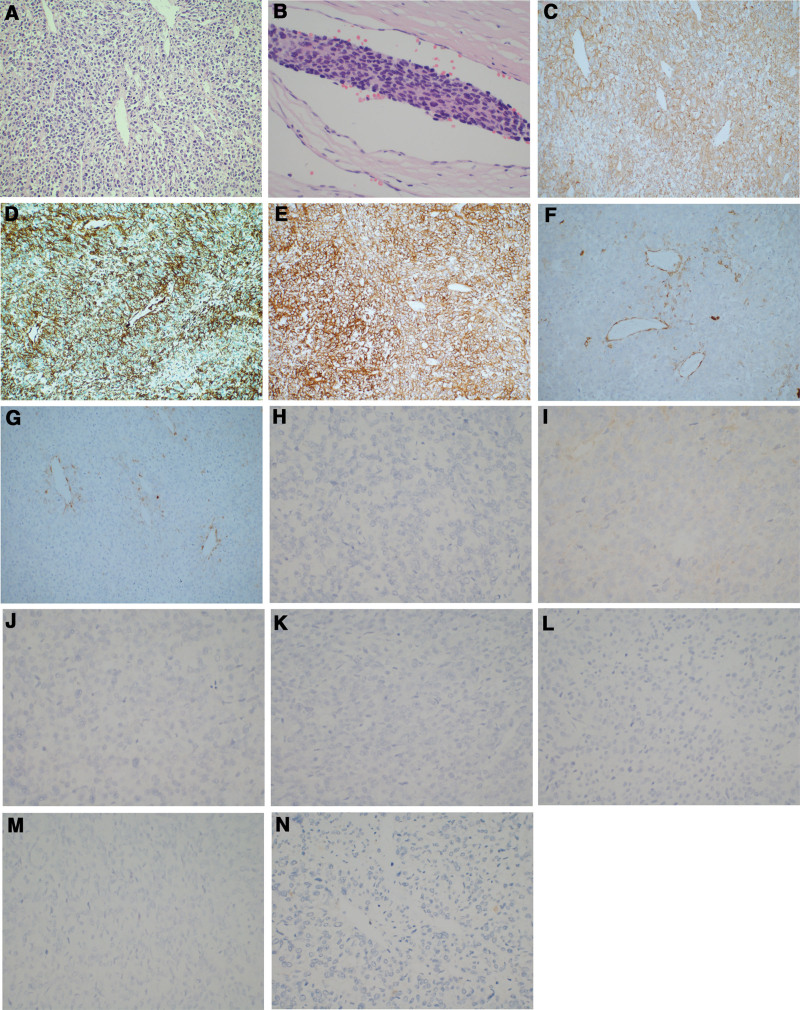
Hematoxylin and eosin (H&E) staining and immunohistochemical staining results. (A) H&E staining showed round cells, arranged in nests around blood channels, moderate-to-severe nuclear atypia (magnification = 200×). (B) Tumor cells with poorly defined, eosinophilic cytoplasm, densely stained chromatin, and moderate-to-severe nuclear atypia were seen in the vascular embolus (magnification = 400×). Immunohistochemical staining showed that the tumor was positive for (C) SMA, (D) vimentin, (E) syn, (F) H-caldesmon (partially positive), (G) calponin (partially positive) (magnification = 200×) while negative for (H) CgA, (I) Dog-1, (J) HMB-45, (K) desmin, (L) S-100, (M) CK-pan, and (N) CD117 (magnification = 400×).

**Figure 3.**
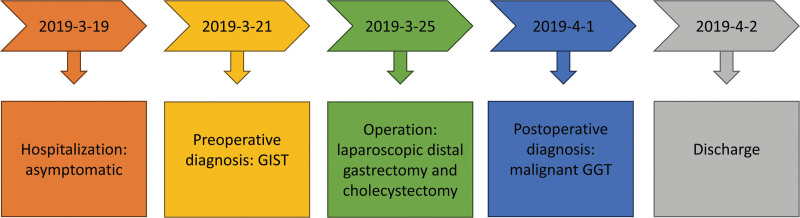
A timeline of this clinical case: the preoperative diagnosis was made by enhanced abdominal CT and ultrasound gastroscopy, and postoperative diagnosis was made by postoperative pathology.

The patient was a healthy male, 49 years old, who was admitted to the First Hospital of Jilin University with multiple gallbladder stones found on a health check. After admission, a CT examination of the abdomen was completed, which showed a mass-like high and low mixed density shadow in the anterior wall of the gastric antrum, with persistent heterogeneous enhancement in the arterial phase, and multiple stones in the gallbladder (Fig. 1A). Ultrasound gastroscopy was then performed, suggesting a hypoechoic mass in the intrinsic muscular layer of the gastric antrum (Fig. 1B and C). The preoperative diagnosis was gastrointestinal stromal tumor (GIST). It was decided to perform laparoscopic distal gastrectomy together with cholecystectomy. After surgery, macroscopic examination revealed a 4.5 cm × 4 cm × 3.5 cm nodular mass in the gastric antrum, grayish and light brown on cut surface, solid and slightly tough. Microscopic examination revealed round cells, arranged in nests around blood channels, moderate-to-severe nuclear atypia, and nuclear mitosis > 10/50 HPF (Fig. 2A). However, atypical mitotic figures were not seen. Vascular invasion was seen but lymph nodes showed no evidence of metastasis. Tumor cells with poorly defined, eosinophilic cytoplasm, densely stained chromatin, and moderate-to-severe nuclear atypia were seen in the vascular embolus (Fig. 2B). Immunohistochemical staining showed that the tumor was positive for SMA, vimentin, syn, H-caldesmon, and calponin while negative for CgA, Dog-1, HMB-45, Desmin, S-100, CK-pan, and CD117 (Fig. 2C–N). The proliferative index (Ki-67) in cellular areas was 20%. The pathological diagnosis was malignant GGT. The patient was satisfied with the treatment and was successfully discharged after 1 week. The patient did not show any discomfort during the 5-year follow-up and no tumor recurrence was seen on gastroscopy and abdominal CT. The timeline of this clinical case is summarized in Figure 3.

## 3. Discussion

We collected almost all well-documented case reports of malignant GGT from PubMed, summarizing their symptoms, immunohistochemistry, surgical methods, follow-up time and outcomes, etc (Table 1).

**Table 1:** Information about case report of malignant GGT.

Author	Gender	Age	Size (cm)	Localization	Symptom
Alsahwan et al^[3]^	Male	56	7 × 4 × 2.5	The proximal part of the greater curvature	Upper GI bleeding
Zaidi and Arafah^[4]^	Female	53	9.7 × 8.8 × 1.1	Gastric fundus	Fullness and pain in the left hypochondrium
Xing et al^[5]^	Female	63	15 × 3	Gastric antrum	Upper abdominal pain
Toti et al^[6]^	Male	72	6 × 4.5	Greater curvature with liver metastases	Hematemesis
Song et al^[7]^	Female	65	3	Gastric fundus with kidney and brain metastases	Dizziness and dyspepsia
Bray et al^[8]^	Male	52	11 × 9 × 17	NA	NA
Davis et al^[9]^	Female	46	1.4	Gastric wall adjacent to the left hepatic lobe margin with liver metastases	Asymptomatic
Folpe et al^[10]^	Male	69	8.5	NA	NA
Teng et al^[11]^	Female	66	5.3 × 5.0 × 4.9	Prepyloric area	Abdominal fullness
Miettinen et al^[12]^	Male	69	6.5 × 6.0 × 3.0	Gastric antrum	NA
Hong et al^[13]^	Male	61	4	Gastric antrum with liver metastases	Dizziness and tarry stool
Present case	Male	49	4.5 × 4 × 3.5	Gastric antrum	Asymptomatic

**Table:** 

Author	Immunohistochemical staining
Alsahwan et al	SMA, vimentin, calponin, synaptophysin(+) CK-pan, CgA, desmin, CD117, Dog-1 and S-100(−) Ki-67(30%)
Zaidi et al	SMA, vimentin, synaptophysin, H-caldesmon, collagen type IV(+) desmin, CD117, S-100, CD34, cytokeratin, HMB-45, chromogranin(−) Ki-67(15%)
Xing et al	SMA(+) CD117, DOG-1, S-100, caldesmon, desmin, CD34(−) Ki-67(30%)
Toti et al	SMA, H-caldesmon, synaptophysin, CD34(+) CK-pan, desmin, DOG-1, S-100, c-kit(−) Ki-67(25%)
Song et al	SMA, synaptophysin, collagen type IV(+) desmin, factor VIII-related antigen, S-100, neurofilament, cytokeratin, and epithelial membrane antigen(−)
Bray et al	SMA, collagen type IV(+) CD117, CD34, CD31, desmin or pancytokeratin(−)
Davis et al	Synaptophysin(+) S-100, CD45, cytokeratin 7, and chromogranin(−)
Folpe et al	NA
Teng et al	SMA, H-caldesmon(+) c-KIT, DOG-1, CD34, cytokeratin, S-100(−)
Miettinen et al	SMA, vimentin, calponin(+) CD117, desmin, S-100, keratin, chromogranin, CD20, CD45(−)
Hong et al	SMA, vimentin, and synaptophysin(+)
Present case	SMA, vimentin, syn, H-caldesmon, calponin(+) CgA, Dog-1, HMB-45, desmin, S-100, CD117, CK-pan(−) Ki-67(20%)

**Table:** 

Author	Mitosis	Operation	Length of follow-up (outcome)
Alsahwan et al	12/10 HPF	Wedge resection of the tumor	6 mo (ANED)
Zaidi et al	10/50 HPF	Resection of the gastric mass	15 mo (ANED)
Xing et al	NA	Distal gastrectomy	16 mo (ANED)
Toti et al	14/10 HPF	Partial gastroresection and Radio Frequency Ablation (RFA)	3 mo (ANED)
Song et al	2/50 HPF	Wedge resection of the stomach and radical nephrectomy	7 mo (DOD)
Bray et al	15/50 HPF	Subtotal gastrectomy	6 yrs (DOD, with scalp, pulmonary, brain, and liver metastases)
Davis et al	NA	Wedge liver biopsy and wedge excision of the stomach tumor	NA
Folpe et al	3/50 HPF	Na	3 yrs (DOD, with liver metastases)
Teng et al	4/50 HPF	Subtotal gastrectomy	9 mo (ANED)
Miettinen et al	1/50 HPF	Na	50 mo (DOD, with liver metastases)
Hong et al	NA	Palliative wedge resection	NA
Present case	>10/50HPF	Subtotal gastrectomy	5 yrs (ANED)

ANED = alive with no evidence of disease, DOD = dead of disease, NA = not available.

**Table:** 

Author	Histological description for the tumor
Alsahwan et al	Moderate nuclear pleomorphism, presence of some atypical mitotic figures
Zaidi et al	Increased cellularity, nuclear atypia, nuclear overlapping, and cuffing of vascular channels by atypical cells
Xing et al	Prominent nucleoli and cellular atypia was seen
Toti et al	More pleomorphic areas with necrosis, nuclear atypia, frequently mitotic activities and focal spindle cell changes were observed
Song et al	Rounded cells with prominent nuclear atypia and some of them were forming multinucleated giant cells
Bray et al	Moderate nuclear pleomorphism and vascular invasion
Davis et al	No mitotic figures or necrosis, but minimal focal calcifications were present within the tumor
Folpe et al	Vascular involvement, but no atypical mitosis or necrosis
Teng et al	Moderate nuclear atypia and vascular invasion was present
Miettinen et al	Mild atypia, and vascular invasion
Hong et al	Nuclear atypia and high mitotic activity
Present case	Moderate-to-severe nuclear atypia, and vascular invasion

As can be summarized from the table, the existing literature showed that the male-to-female ratio of malignant GGT was about 1.4:1, the average age was (60 ± 8) years, and the length diameter of the tumor was (7.3 ± 4.5) cm. Patients’ symptoms were mainly upper gastrointestinal bleeding and upper abdominal pain, some patients could be asymptomatic. A few patients were found to have liver, kidney, or even brain metastases at the time of detection.

The preoperative diagnosis of malignant GGT is comparatively difficult, and most cases are only clearly diagnosed by postoperative pathology, among which there are many cases of preoperative misdiagnosis as GIST.^[11]^ Enhanced abdominal CT and ultrasound gastroscopy are necessary preoperative examinations, but GGT and GIST both originate from the intrinsic base of the stomach and are challenging to differentiate on imaging. Xing et al^[5]^ compared 21 cases of GGT with 30 cases of GIST and 30 cases of heterotopic pancreas, and concluded that multi-phase CT enhancement usually shows persistent and significant enhancement, especially in the arterial phase, which is of great value for the diagnosis of GGT. Wang et al^[15]^ compared the CT images of 11 cases of GGT with 48 cases of GIST, concluded that the following 7 clinical and imaging features could help in the diagnosis of GGT: location in the antrum, endophytic growth, heterogeneous enhancement in the arterial phase, CT value in the arterial phase of ≥60.7 HU, CT value in the portal venous phase of ≥87.6 HU, degree of enhancement (arterial phase) of ≥29.9 HU, and degree of enhancement (portal venous phase) of ≥49.0 HU. In the present case, CT of the tumor in the gastric antrum showed persistent heterogeneous enhancement in the arterial phase, which is mostly absent in GIST. Actually, there were evidences to consider the possibility of GGT, it was just the lack of attention to these details and stereotype that led to the preoperative misdiagnosis. Akahoshi et al^[16]^ performed endoscopic ultrasound-guided fine needle aspiration (EUS-FNA) in 47 cases of gastric subepithelial lesion < 2 cm, including a GGT, which was clearly diagnosed preoperatively. The diagnostic accuracy of EUS-FNA using immunohistochemical analysis was 98%, and none of the tumors recurred after surgery. Thus, EUS-FNA is an accurate and safe method for preoperative diagnosis of gastric subepithelial lesions smaller than 2 cm, and it is a reliable method for those who seek a definitive preoperative diagnosis. In this case, the patient’s tumor was larger than 2 cm, the safety and accuracy of EUS-FNA is not supported in the literature, so trying to make a definitive diagnosis preoperatively is almost too risky and improbable to accomplish. Experienced surgeons can make an initial differential diagnosis by preoperative examinations, including enhanced CT and ultrasound gastroscopy, in conjunction with the above points. And further differential diagnosis can be made by intraoperative frozen section. However, intraoperative frozen section does not make a definitive diagnosis and does not change the surgical approach, so its significance is limited.

Malignant GGT is solid tumor, mostly gray or light brown in cross-section, slightly tough in texture, and may have single or multiple cystic structures.^[4]^ No lymph node metastasis was found in previous reports, while several reports documented vascular invasion of the tumor. Distant metastasis of the tumor was are most commonly found in the liver.^[12]^ Our case was also consistent with previous literature, with the presence of vascular invasion without lymph node metastasis, and we showed microscopic photographs of vascular invasion of the tumor, with moderately-to-severe nuclear atypia of tumor cells within the embolus. Based on the tumor morphology and biological characteristics, it is reasonable to speculate that malignant GGT metastasizes mainly by hematogenous route. Malignant GGT microscopically showed nodular or nest-like arrangement of tumor cells, the nests of cells were separated from the nodules by smooth muscle bundles, the tumor was rich in blood vessels, and the cells surrounded the blood vessels in a vascular epithelioma-like structure. Five of the 12 cases of malignant GGT listed in the table had a mitotic count ≥ 10/50 HPF, 4 cases < 5/50 HPF, and 2 cases had no mitotic count mentioned. Immunohistochemical staining did not differ between benign and malignant tumors, with positive results for SMA, vimentin, synaptophysin, H-caldesmon, collagen type IV, calponin and negative results for CK-pan, CgA, desmin, CD117, Dog-1, and S-100.^[17]^ The Ki-67 index is around 20% to 30%. Mosquera et al^[18]^ performed RNA sequencing on 5 malignant and 28 benign GT and found that all 5 malignant GT showed rearrangements of NOTCH2, with 3 of them being fused to MIR143, which included a case of malignant GGT. Papke et al^[19]^ performed next-generation DNA sequencing of 10 malignant and 5 benign GGT, found that NOTCH2 alterations occurred in 80% of GGT, and all 5 cases of benign GGT lacked complex copy number alterations (CNAs), whereas 10 cases of malignant GGT showed complex CNAs.

The surgical treatment of malignant GGT is based on negative margin surgery, including subtotal gastrectomy and wedge resection of the stomach. We report the first case of malignant GGT in the presence of vascular invasion without recurrence during 5 years of postoperative follow-up, so malignant GGT without distant metastases can have a good prognosis after surgical resection. There is no report on the effectiveness of radiotherapy and chemotherapy. Patients found to have distant metastases had a poor prognosis after surgical resection, but in one case there was no sign of recurrence at 3-month follow-up after resection of liver metastases, partial gastroresection, and radio frequency ablation.^[6]^ Xu et al^[20]^ reported a 3.8 × 3.1 cm GGT treated by endoscopic submucosal dissection (ESD) with complete resection of the tumor, but the article did not report prognostic information. ESD may be a treatment modality for small GGT, but its safety and efficacy need to be validated in multiple centers. Deng et al^[21]^ analyzed the prognosis of 31 patients with GGT, concluded that tumor diameter ≥ 5 cm was associated with mortality outcome of GGT, severe cellular atypia, and atypical mitosis were associated with poor prognosis of GGT. However, Davis et al^[9]^ reported a case of malignant GGT with liver metastasis with a primary lesion of only 1.4 cm in diameter. Song et al^[7]^ reported a case of malignant GGT of 3 cm in diameter with renal and cranial metastases, whose atypical mitosis was only 2/50 HPF. Miettinen et al^[12]^ also reported a case of malignant GGT with atypical mitosis of only 1/50 HPF, who died 50 months after diagnosis due to the development of liver metastases. This leads to a rethinking of the determination of malignant GGT. The definition of malignant glomus tumor was first proposed by Folpe et al^[10]^ as malignant glomus tumors are tumors with a deep location and a size of more than 2 cm, or atypical mitotic figures, or moderate to high nuclear grade and ≥5 mitotic figures/50 HPF. Then Papke et al^[19]^ analyzed 26 cases of GGT and proposed the hypothesis for the determination of malignant GGT, cytologic atypia and ≥2 mitoses/10 HPF; or tumor diameter ≥ 5 cm. In the author’s opinion, although many GGTs are larger than 2 cm, we may define malignant GGT by the criteria of Folpe et al, at the same time, cytologic atypia and ≥2 mitoses/10 HPF, tumor length diameter ≥ 5 cm, complex CNAs, as well as vascular invasion can be used as poor prognostic factors. Although a good prognosis can be obtained with negative margin surgery, these patients with poor prognostic factors should be followed up more closely after surgery to avoid tumor recurrence and metastasis.

In conclusion, we reported a case of malignant GGT, the patient was admitted with gallbladder stones but CT revealed a mass in the gastric antrum, so we performed laparoscopic distal gastrectomy together with cholecystectomy. Postoperative pathology suggested malignant GGT with vascular invasion. The patient discharged 1 week after operation and no recurrence of the tumor was seen during the 5-year postoperative follow-up. So, malignant GGT can be asymptomatic. For malignant GGT without distant metastasis, despite the presence of vascular invasion, negative margin surgery can still be the standard surgical radical treatment.

## Author contributions

**Writing – original draft:** Jiannan Huang.

**Writing – review & editing:** Jiannan Huang, Chaofeng Yuan, Jian Suo.

**Resources:** Shaopeng Zhang, Tong Qu.
